# A bioinspired bubble removal method in microchannels based on angiosperm xylem embolism repair

**DOI:** 10.1038/s41378-022-00367-1

**Published:** 2022-03-22

**Authors:** Lihua Guo, Yuanchang Liu, Penghui Ran, Gang Wang, Jie Shan, Xudong Li, Chong Liu, Jingmin Li

**Affiliations:** 1 Key Laboratory for Micro/Nano Technology and System of Liaoning Province, Dalian University of Technology, Dalian, China; 2 Department of Mechanical Engineering, University College London, Torrington Place, London, WC1E 7JE UK

**Keywords:** Electrical and electronic engineering, Chemistry

## Abstract

It is difficult to remove and eliminate bubbles in microchannels in many devices used in various biomedical fields, such as those needed for microfluidic immunoassays, point-of-care testing, and cell biology evaluations. Accumulated bubbles are associated with a number of negative outcomes, including a decrease in device sensitivity, inaccuracy of analysis results, and even functional failure. Xylem conduits of angiosperm have the ability to remove bubbles in obstructed conduits. Inspired by such an embolism repair mechanism, this paper proposes a bioinspired bubble removal method, which exhibits a prominent ability to dissolve bubbles continuously within a large range of flow rates (2 µL/min–850 µL/min) while retaining the stability and continuity of the flow without auxiliary equipment. Such a method also shows significant bubble removal stability in dealing with Newtonian liquids and non-Newtonian fluids, especially with high viscosity (6.76 Pa s) and low velocity (152 nL/min). Such advantages associated with the proposed bioinspired method reveal promising application prospects in macro/microfluidic fields ranging from 3D printing, implantable devices, virus detection, and biomedical fluid processing to microscale reactor operation and beyond.

## Introduction

Because of the features of small size, low sample consumption, and fast response with a high surface-to-volume ratio, microfluidics has wide application prospects in biomedical fields, offering significant advantages over conventional methods. The microfluidic system always integrates complex architectures of interconnecting pumping systems, fluid delivery channels, micromixers, and detection elements to execute a variety of tasks where stable flow delivery is critical. The formation and accumulation of bubbles in microchannels is a ubiquitous problem in biomedical fields, especially in microfluidic immunoassays^1^, point-of-care testing^2^, and cell biology^3^. Accumulated bubbles result in poor reaction performance, a decrease in device sensitivity, inaccurate analysis results, and even functional failures. For instance, nucleation and adhesion of bubbles on the surface of an immunoassay device reduces the size of the effective reaction areas, causing an insufficient quantity of reactants and nonspecific binding^4,5^, which leads to low device sensitivity and, subsequently, incorrect results. Surface tension at the gas–liquid interface can not only cause irreparable damage to the chemical grafting of antibodies and surface modification of nanoparticles in point-of-care devices but can also hinder the supply of reagents by obstructing fluidic paths and lead to pressure fluctuations. Therefore, removing bubbles in an automatic steady way in real time is of great importance in microfluids.

At the moment, dominant approaches of bubble removal in microchannels include passive and active methods. Passive methods, including increasing inlet pressure^5^, flushing microchannels with low-polarity aqueous solutions^6,7^, treating surfaces with hydrophilic modification methods^4,8^, and integrating bubble trap structures^9,10^ on chips, have been used to prevent bubble formation and injection. However, increasing the inlet pressure, flushing microchannels, and hydrophilic modification cannot remove bubbles that form during the experiment. Bubble-trap structures are constrained by the drawback of incapacity in dealing with a myriad of bubbles due to limited trapping volumes. To address these issues, active methods have been developed to capture and extract trapped bubbles, and these methods can be categorized into the gas–liquid separation method based on surface treatment^11–13^ and the bubble extraction method based on hydrophobic porous membranes^14–18^. However, the gas–liquid separation method is time-dependent and poor in biocompatibility. The bubble extraction method has the advantage of a high bubble removal rate, but there are some drawbacks that limit its application. First, the bubble extraction method cannot be used in liquid with high viscosity, liquid with high pressure or high flow rate, or gas impermeable material. High viscosity fluids have a thick boundary layer that prevents gas permeation through the membrane. The bubble extraction method is not suitable for bubble removal of high viscosity fluids. In addition, a pressure and flow rate that is higher than a critical value will cause liquid to leak from the porous hydrophobic membrane^19^. Moreover, the bubble extraction method relies on the hydrophobicity of the material to trap bubbles and the gas permeability of the material to extract air bubbles from the liquid. It cannot be used in the situation where hydrophilic modification of the microchannel wall is required and the situation where microfluidic chips are used in liquid environments, such as implantable devices and water quality detection devices. Second, the bubble extraction method requires additional vacuum pressure and a multilayered structure, both of which are relatively difficult to fabricate and inconvenient to integrate.

Attention can therefore be cast on natural designs, where angiosperms use pits as sentinels to remove bubbles in xylem conduits formed in complicated environments. Such a process is also known as embolism repair^20–22^. Pits with micro- and nanometer-sized pores can prevent air from spreading between adjacent conduits^23,24^ and provide auxiliary hydraulic channels when conduits are blocked by bubbles^25,26^. The pressure difference generated around the captured bubble can increase the solubility of gas to liquid, and continuous water flowing around bubbles accelerates the dissolution of bubbles. It has been demonstrated that xylem conduits can remove bubbles within several minutes without causing extra external damage^27,28^.

Therefore, inspired by such a promising bubble removal mechanism that resides in xylem conduits, a bioinspired bubble removal method (BBR) has been proposed and designed in this paper. Redundant channels mimicking pits of angiosperm were designed to maintain the flow continuity and subsequently remove bubbles. To verify the performance of bubble removal, a series of tests were conducted using the device based on the BBR method. The results demonstrate that the time taken by the bubble removal process is related to the geometric parameters of the bionic structures, flow rate, and viscosity. In particular, configurations with a high flow rate, small pits, and narrow channels contribute to a short bubble removal process time.

In addition, microchannel arrays based on the BBR method were designed to verify the reliability of removing continuous bubbles within a large range of flow rates, i.e., in Newtonian liquids and non-Newtonian liquids with high viscosity. Furthermore, the test results reveal that using the proposed BBR method, the dissolution of bubbles can be achieved in a rapid way without using any other auxiliary equipment. Bubbles can be removed by retaining the stability and continuity of the flow, which can be applied to devices made of a variety of materials.

Compared to the characteristics and performance of the existing bubble removal methods, the BBR method proposed in this work has the following advantages. First, it has a wide range of applications. The BBR method can remove bubbles in both high viscosity fluids and regular fluids. It could also remove bubbles in liquid with high pressure (when dealing with high viscosity fluid) and high flow rate (by increasing the channel number and adjusting the structural parameters). The BBR method can adjust the bubble removal rate and bubble removal amount to meet the requirements of different environments by changing its geometric parameters and channel number. Thus, the BBR method has a wide range of applications in the microfluidic field, including removing bubbles in high viscosity liquid (such as gels), removing bubbles in the situation where hydrophilic modification of the microchannel wall is required and the situation where microfluidic chips are used in liquid environments (such as implantable devices and online water quality detection devices). Second, the BBR method is not permeable material-dependent and has no material restrictions. The BBR method does not rely on the hydrophobicity of the material to trap bubbles and the gas permeability of the material to extract air bubbles. The BBR method removes bubbles based on the bionic mechanism that resides in xylem conduits. Bubbles are captured by pits and dissolved in liquid gradually by using the pressure difference generated around the captured bubble. Therefore, there is no limitation for the material to fabricate the device by using this method. The BBR method can be used for both hydrophobic materials (such as PDMS and PMMA) and hydrophilic materials (such as silicon and glass). Third, the BBR method is easy to fabricate and integrate. The bioinspired bubble removal unit has two layers, and only one layer needs to be photolithographed. In addition, this method could remove bubbles without additional vacuum equipment. It is easy to fabricate and integrate into various devices according to the application requirements. Fourth, the BBR method could remove bubbles in an automatic, real-time, and steady way. The BBR method can remove bubbles automatically and continuously in real time without the aid of human interference and external equipment.

A detailed comparison of different bubble removal methods is summarized in the supplementary information and Table S1.

## Results

### The bionic mechanism of bubble removal

**Fig. 1:**
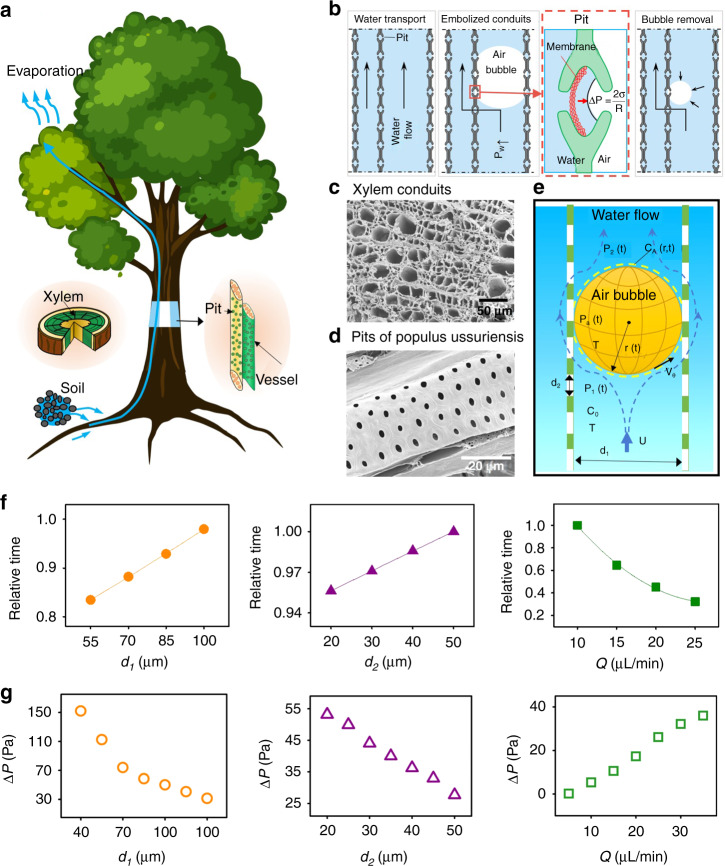
Schematic representation of embolism repair in angiosperm. **a** Water transport in xylem from soil to leaves is driven by evaporation in the daytime and root pressure at night. The xylem conduit in angiosperm is also called a vessel, whose diameter ranges from tens to hundreds of micrometers. **b** Air bubbles in vessels can inflate and block water transportation to form air embolisms. A curved gas-water interface in the pit can be generated as bubbles inflate to prevent air bubbles from expanding to adjacent conduits. When water flow encounters a stuck bubble, it will pass through nearby pits and enter adjacent conduits to continue the water transportation. As water flows around bubbles and removes the dissolved air, a gradual bubble dissolution process occurs until all bubbles disappear. **c**, **d** SEM images of Populus ussuriensis xylem conduits and pits. **e** A dissolution model of bubbles was established to explicate the bubble removal process. **f** Prediction results of bubble relative removal time (the ratio of each bubble removal time to the maximum) with respect to pit size *d*_2_, conduit diameter *d*_1_ and flow rate *Q*. **g** Simulation results of the pressure difference between the head and tail of a bubble with respect to pit size *d*_2_, conduit diameter *d*_1_ and flow rate *Q*

In plants, water transportation from root to leaf can be explained by the cohesion-tension theory^29^, where a negative pressure generated by leaf evaporation and the root pressure drives the water upward (Fig. 1a). In some environmental conditions, such as drought or freezing, negative pressure can desaturate the gas in water, which results in the generation and inflation of bubbles^22,30,31^. At night, as root pressure dominates water transportation, the negative pressure weakens, and the air solubility in water increases^32^. As a consequence, bubbles are removed, and conduits are refilled^33^.

Figure 1b schematically illustrates the mechanism of embolism recovery in xylem vessels. Air bubbles in vessels can inflate and block water transportation. Pits are essential features to prevent air bubbles from expanding to adjacent conduits and maintain flow continuity. A curved gas-water interface in the pit can be generated as bubbles inflate. According to the Young–Laplace equation, the pressure difference Δ*P*_*a*_ that is sufficient to isolate neighbor conduits is

1 $$\Delta P_a = \frac{{2\sigma }}{{R_c}}$$

where *R*_*c*_ is the radius of curvature and *σ* is the surface tension force. Figure 1c, d show scanning electron microscopic (SEM) images of the xylem conduits and the pits of Populus ussuriensis, respectively.

The principal role of pits is to provide auxiliary hydraulic channels when xylem channels are blocked. When water flow encounters a stuck bubble, it will pass through nearby pits and enter adjacent conduits to continue the water transportation. In this process, water in plants can be described as incompressible Newtonian liquids. The narrow xylem conduits combined with low flow velocity *U* result in a small Reynolds number ($0.004 \le R_e \le 3.985$). Further simplifications can be obtained by treating water as a crawling flow around a spherical bubble. The water around the bubble can be described by the Navier–Stokes equation in the form of a flow function in a spherical coordinate system (Fig. 1e) as:

2 $$\left[ {\frac{{\partial ^2}}{{\partial r^2}} + \frac{{\sin \theta }}{{r^2}} \times \frac{\partial }{{\partial \theta }}\left( {\frac{1}{{\sin \theta }} \times \frac{\partial }{{\partial \theta }}} \right)} \right]^2\psi = 0$$

where *r* is the radial distance from the bubble center, *θ* is the azimuth angle in the spherical coordinate system, and *ψ* is the flow function. Under the condition of constant temperature *T* and pressure, the concentration field of air dissolved in water is $c_A\left( {r,t} \right)$, and the solubility of air in water is *c*_*A*1_ and *c*_*A*2_ at the head and tail of the bubble, respectively. The equilibrium concentration of gas dissolved in water outside the concentration boundary layer is *c*_0_. The conservation of mass of water flow can be described by the continuity equation as:

3 $$\frac{1}{{r\sin \theta }} \times \left( {\frac{{\partial \left( {v_\theta \sin \theta } \right)}}{{\partial \theta }}} \right) = 0$$

Due to the small diffusion coefficient of air in flowing water, the concentration boundary layer is relatively thin compared with the diameter of the bubble. Hence, it is reasonable to consider that the velocity field is not affected by a gas–liquid mass transfer and that the concentration field in water is constant. The mass transfer process can be described by the advection-diffusion equation as:

4 $$v_\theta \frac{{\partial c_{{{\mathrm{A}}}}}}{{\partial r}} = D_{{{{\mathrm{AB}}}}}\frac{1}{{r^2}}\frac{\partial }{{\partial r}}\left( {r^2\frac{{\partial c_{{{\mathrm{A}}}}}}{{\partial r}}} \right)$$

where *D*_*AB*_ is the diffusion coefficient and *c*_*A*_ is the concentration field of air dissolved in water. Combining Eqs. (2), (3), (4) and according to Fick’s first law, the conservation of mass, Henry’s law, and the equation of state for ideal gas, finally, we can calculate the change rate of bubble radius *R* as:

5 $$\frac{{dR}}{{dt}} = - \Re T\left( {K_H - \frac{{2c_0}}{{2P_2 + \Delta P + 2\sigma /R}}} \right)\sqrt {\frac{{2D_{AB}U}}{{3\pi R}}}$$

where *t* is the time variable, $\Re$ denotes the universal gas constant, *K*_*H*_ is Henry’s coefficient, *P*_2_ is the pressure at the tail of the bubble, and Δ*P* is the pressure difference between the head and tail of a bubble, which is affected by the structural parameters of the pit and bubble radius as:

6 $$\Delta P = P_1 - P_2{{{\mathrm{ = }}}}0.71\rho _w\left( {1 - \frac{{d_{{{\mathrm{2}}}}^2}}{{d_{{{\mathrm{1}}}}^2}}} \right)\left( {\frac{{4Q}}{{\pi d_2^2}}} \right) + \rho _wK\frac{{v_\theta ^2}}{2}$$

where *P*_1_ is the pressure at the head of the bubble, *ρ*_*w*_ is the water density, and *d*_1_ and *d*_2_ are the diameters of the conduit and pit, respectively. *Q* is the flow rate in the conduit, and *K* is the coefficient depending on the bubble radius and adjacent conduits.

To verify the proposed bionic mechanism, a numerical simulation was conducted by using ANSYS FLUENT software. The result is shown in Fig. S1. Air bubbles are shown in red, while water is shown in blue. During the simulation, when a bubble entered the xylem conduit, it was captured by pits. Water around the bubble passed through nearby pits and entered adjacent conduits to continue water transportation. The simulation results show that pits provide auxiliary hydraulic channels when the conduit is blocked. Fig. S1b shows the pathlines of water in the conduit when it was blocked by bubbles. Then, the bubble was eliminated gradually when water bypassed it (Fig. S1c). During the process of bubble elimination, pits prevented air bubbles from expanding to adjacent conduits. The theoretical and simulated bubble removal times are compared in Fig. S1d. The theoretical bubble removal time (calculated by using Eq. 5) is in agreement with the simulation time. The error between the simulation and theoretical model occurred because the bubbles were simplified to an ideal spherical shape in the theoretical model, while the bubbles did not have an ideal spherical shape in the simulation. Figure 1g shows the relationship between the pressure difference and pit size, conduit diameter, and flow rate. With increasing pit size and conduit diameter, the pressure difference decreases. As the flow rate increases, the pressure difference increases.

From Eqs. (5) and (6) and the simulation, important statements can be reached:

1. As water flows around bubbles and removes the dissolved air, a gradual bubble dissolution process occurs until all bubbles disappear;
2. The increased pressure difference around the captured bubble can improve the bubble removal rate. In addition, a high flow velocity *U* can facilitate bubble removal;
3. The bubble removal rate is affected by the pit size *d*_2_, conduit diameter *d*_1_, and flow rate Q (as graphically shown in Fig. 1f). A smaller pit, a smaller pit-to-conduit diameter ratio, and a faster flow rate can generate a higher pressure difference, which consequently can increase the bubble removal rate.

### Design of the bubble removal device based on the BBR method

**Fig. 2:**
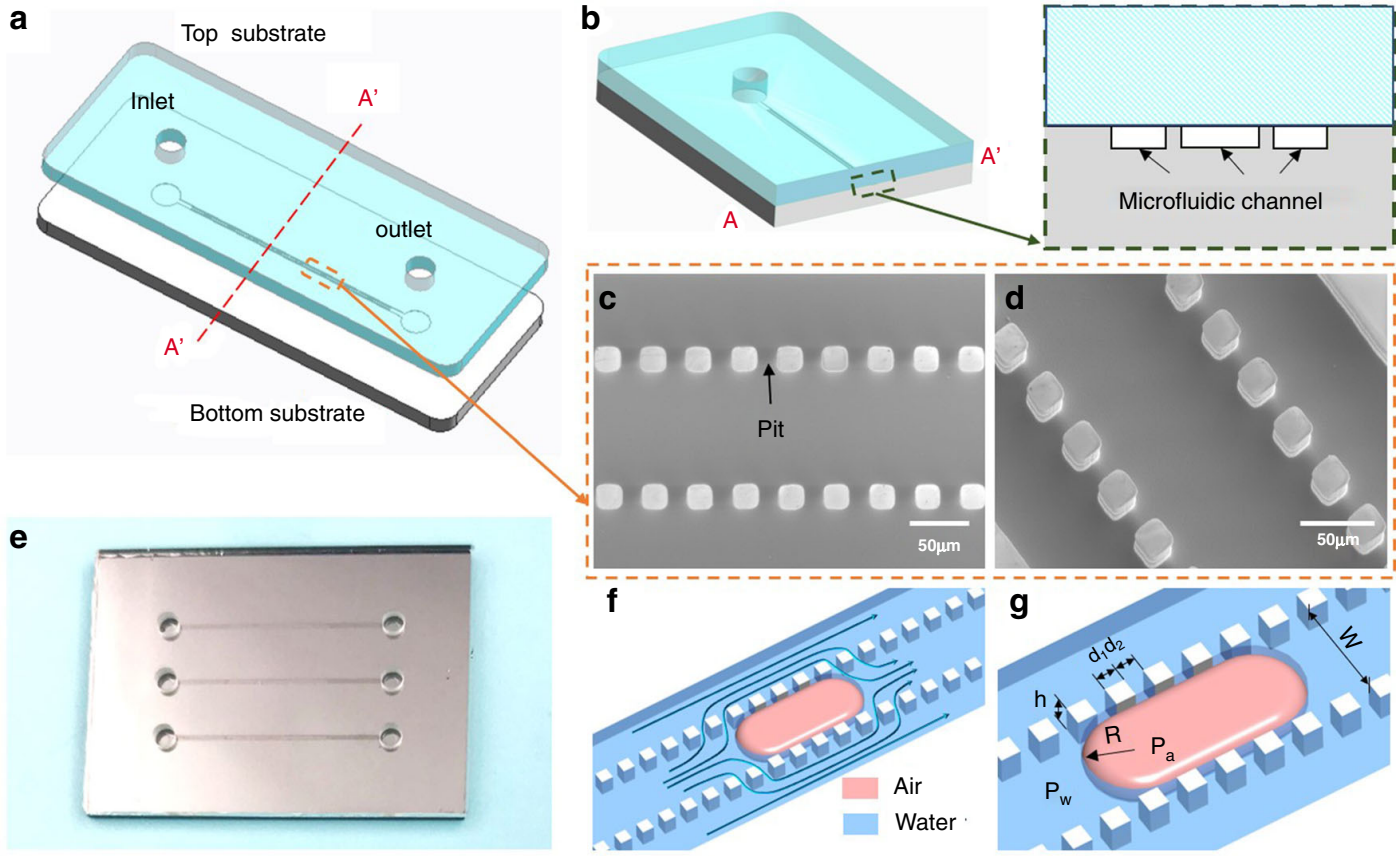
Bubble removal device inspired by the bubble removal mechanism of angiosperm xylem. **a** The bubble removal device is composed of a glass cover and a bottom substrate. The glass cover contains inlets and outlets. **b** There are three bioinspired microchannels on the bottom substrate and pits to make the channels interconnect with each other. **c**, **d** Scanning electron microscope (SEM) images of the bioinspired microchannels. **e** Photograph of an assembly bioinspired bubble removal device. **f**, **g** An air bubble is captured by pits. The fluid flows around the bubble. The width of the middle channel is *w*, the pit size is *d*_*1*_, the pit interval is *d*_*2*_, the pit height is *h*, and the bubble radius is *R*

A bubble removal device based on the BBR method is composed of a bottom silicon substrate and a top glass cover (Fig. 2a). The bottom silicon substrate is designed with microchannels and rows of pits to interconnect them (Fig. 2b, c, d). The inlet and outlet are placed on the top glass cover. When a bubble flows into the device from the inlet, it will be trapped by pits. When a microchannel is blocked by a bubble and becomes dysfunctional, water in this channel can continue to flow and bypass the bubble through pits (Fig. 2f, g). When such a scenario happens, on the one hand, these pits function as bubble trappers that can capture bubbles in flowing water. On the other hand, the designed pits prevent bubbles from flowing into other microchannels and provide a sideway to transport water. As the process continues, the bubble can be dissolved gradually when water bypasses around it.

### Effects of geometric parameters of bionic structures, flow rate and liquid viscosity

**Fig. 3:**
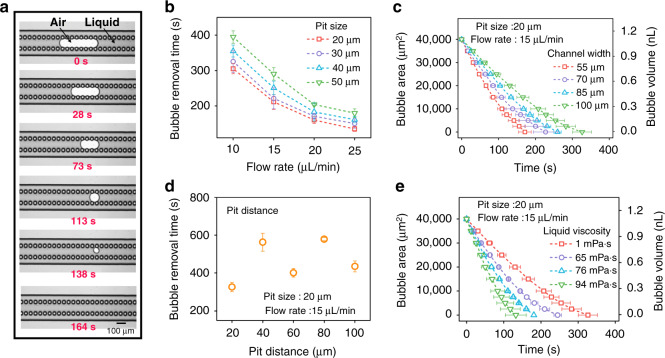
Removal performance of the bubble removal device. **a** The bioinspired bubble removal device is capable of eliminating bubbles in microchannels. **b** Variation in bubble removal time with respect to the flow rate (10 µL/min, 15 µL/min, 20 µL/min, 25 µL/min) and pit size (20 µm, 30 µm, 40 µm, 50 µm). **c** Variation in the bubble removal time (171 ± 17 s, 227 ± 33 s, 260 ± 10 s, 326 ± 25 s) with respect to channel widths of 55 µm, 70 µm, 85 µm and 100 µm (pit size of the bubble removal device is 20 µm, pit distance is 20 µm and flow rate is 15 µL/min). **d** Variation in the bubble removal time with respect to pit distances of 20 µm, 30 µm, 40 µm and 50 µm (pit size of the bubble removal device is 20 µm, the channel width is 100 µm and flow rate is 15 µL/min). **e** Variation of the bubble removal time (326 ± 25 s, 245 ± 10 s, 181 ± 3 s and 132 ± 28 s) with respect to the liquid viscosity of 1 mPa·s, 65.38 mPa·s, 75.62 mPa·s and 93.57 mPa·s at 25°C (pit size of the bubble removal device is 20 µm, channel width is 100 µm and flow rate is 15 µL/min)

To verify the bubble removal ability of the BBR method, the device was injected with water containing bubbles (Fig. 3a and Movie S1). The flow rate was 20 µL/min, and the volume of the air bubble was 1.2 nL. From the top view, it could be observed that the air bubble was captured by pits after a short slide. Subsequently, the captured air bubble was dissolved gradually until it was removed. To verify the essential role of the pits in the process of bubble removal, the bubble behavior in the microchannel without pits was also tested. The width and length of the channel are the same as those of the channel integrated with pits. The flow rate was 20 µL/min. The results show that the bubble was either flushed out or stuck, which blocked the channel (Movie S2).

The geometric parameters of bionic structures, including pit size, channel width, and pit distance, are crucial factors that determine the bubble removal time. To study the bubble removal effect of the device, a series of bubble removal devices based on the BBR method were fabricated (Fig. S3). Figure 3b shows how the bubble removal time varies with respect to pit size. For example, with a flow rate of 20 µL/min, the bubble removal times under pit sizes of 20 µm, 30 µm, 40 µm, and 50 µm are 160 ± 10 s, 171 ± 8 s, 183 ± 25 s and 204 ± 3 s, respectively. It can be summarized that a small pit size results in a short bubble removal time. Channel width also plays a critical role in bubble removal. As shown in Fig. 3c, in the bubble removal device with a pit size of 20 µm, a pit distance of 20 µm and a flow rate of 15 µL/min, the bubble removal times with channel widths of 55 µm, 70 µm, 85 µm, and 100 µm are 171 ± 17 s, 227 ± 33 s, 260 ± 10 s and 326 ± 25 s, respectively. This indicates that a narrow channel width contributes to a short bubble removal time.

Compared with the clear variation tendency of pit size and channel width, the relationship between the pit distance and removal time is relatively complicated (Fig. 3d). For instance, in the bubble removal device with a pit size of 20 µm and a flow rate of 15 µL/min, the removal times with pit distances of 20 µm, 40 µm, 60 µm, 80 µm, and 100 µm are 326 ± 25 s, 563 ± 47 s, 401 ± 22 s, 579 ± 8 s and 435 ± 29 s, respectively. It can be observed that pit distance can also affect the removal time, but there is no obvious linear relationship.

In addition to the appropriate geometric parameters of bionic structures, increasing the flow rate is also beneficial to reduce the bubble removal time (Fig. 3b). For example, in the bubble removal device with a pit size of 20 µm, the bubble removal times with flow velocities of 10 µL/min, 15 µL/min, 20 µL/min and 25 µL/min are 305 ± 14 s, 211 ± 21 s, 160 ± 10 s and 135 ± 7 s, respectively.

Liquid viscosity is also an evident factor that can affect the bubble removal time (Fig. 3e). For the liquids with viscosities of 1 mPa s, 65.38 mPa s, 75.62 mPa s and 93.57 mPa s at 25 °C, the bubble removal times are 326 ± 25 s, 245 ± 10 s, 181 ± 3 s and 132 ± 28 s, respectively. With increasing liquid viscosity, the bubble removal time decreases.

Moreover, the pits in the channel have the capability of filtering undesired particles/objects. When impurities or particles are stuck in a pit, the pits nearby can provide redundant channels, and the liquid can still flow and remove the bubbles. An experiment was performed to verify that the device can be reused for bubble removal and liquid transportation. Movie S3 shows the bubble removal process when impurities or particles are trapped in the channel. The bubble removal device based on the BBR method can work normally as well.

### The flow rate range applicable for removing bubbles of the BBR method

**Fig. 4:**
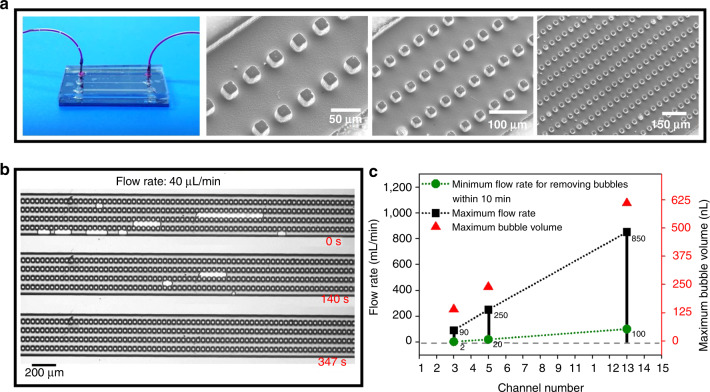
Bubble removal performance of microchannel arrays. **a** Photograph and SEM images of microchannel arrays. **b** The bubble removal device with five microchannels based on the BBR method is capable of eliminating continuous bubbles. **c** Variation in the flow rate with respect to the number of microchannels

The BBR method has the capability of removing bubbles within a large range of flow rates. As shown in Fig. 4a, pink ink mixed with continuous bubbles was injected into the bubble removal device with a pit size of 20 µm, a channel width of 70 µm, and channel numbers of 3, 5, and 13 via a T-junction bubble formation device. The first injected bubble slipping in the channel was captured by pits. While the first bubble is being removed, the later bubble either slips into another unblocked microchannel or encounters the first bubble ahead and merges into a larger one. Then, the merged bubble was stopped by pits and dissolved gradually until it disappeared (Fig. 4b and Movie S4). Through such an iterative process of merging (or capture) and elimination, the injected bubbles can be effectively and efficiently removed in microchannels, which makes the designed bubble removal device capable of capturing and eliminating continuous bubbles.

Furthermore, the maximum bubble volumes that the bubble removal device with channel numbers of 3, 5, and 13 is able to remove at one time are 140 nL, 239 nL, and 610 nL, respectively. When the fluid in microchannels moves with a high velocity, bubbles injected in the microchannels can slip out, leading to a potential function failure of the device. Consequently, the performance of the proposed bubble removal device is constrained by a maximum flow rate, which is associated with a specific volume. As shown in Fig. 4c, in the bubble removal device with 3, 5 and 13 channels, the maximum flow rates for capturing and eliminating continuous bubbles are 90 µL/min, 250 µL/min, and 850 µL/min, respectively. The minimum flow rates for eliminating bubbles within 10 min are 2 µL/min, 20 µL/min, and 100 µL/min. In addition, the maximum and minimum flow rates with respect to the number of channels have a linear relationship. To remove bubbles with a low flow rate, narrow channels can be adopted in the designed bubble removal device, whereas to remove bubbles with a high flow rate, multiple wide channels can be implemented. Therefore, the BBR method can be applied to bubble elimination with a large range of flow rates.

### BBR method used in Newtonian liquids

Liquids such as chemical stains and biological medicine used in biochemical analysis tend to generate bubbles in microchannels, especially in complicated structures. A concentration gradient generator was fabricated to verify the capacity of the proposed BBR method in dealing with Newtonian liquids. Concentration gradient generators are widely used in biological processes such as drug screening, cell growth and chemotaxis. Bubbles in the fluid channels will lead to the problem of nonuniform drug distribution, and we show that this problem can be effectively solved by using the proposed bubble removal device.

**Fig. 5:**
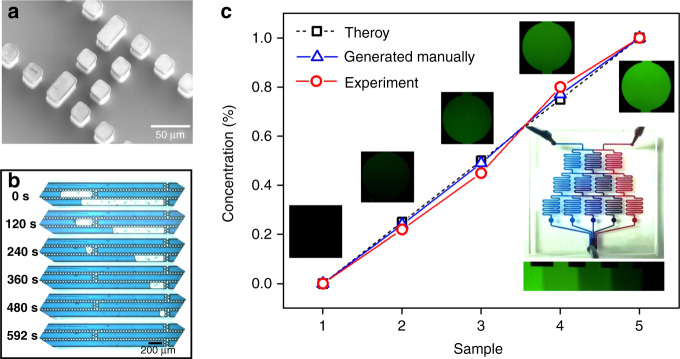
Application of a bubble removal device in a concentration gradient generator. **a** SEM image of bubble trappers in the channel. **b** The bubble removal device could be capable of eliminating bubbles before blue ink flowed into the concentration gradient generator. **c** Both ink and fluorescein flow through the bubble removal device before the concentration gradient generator. The plot indicates the theoretical and experimental concentrations of fluorescence in the collecting reservoir. The micrographs show the fluorescence intensity with respect to the concentration

To improve the bubble capture ability, the proposed bubble removal device has been integrated with bubble trappers in the microchannels (Fig. 5a). To test its performance, a bubble removal device was connected to the inlet of a concentration gradient generator (Fig. S7). To compare the results, experiments were also conducted on a concentration gradient generator without bubble removal devices (Fig. S8). In the experiment when bubble removal devices are available, the bubbles in the fluid can be well captured by bubble trappers and eliminated by the bubble removal devices (Fig. 5b). Finally, the bubble-free ink diffused, and ink with five different concentrations was produced. To measure the concentration distribution, the same experiment was carried out with deionized water and sodium fluorescein solution (100 μM). The brightness of the mixed solution with different concentrations was characterized by using an inverted microscope and compared with the theoretical value. To validate the results, standard concentrations of sodium fluorescein solution (0 μM, 25 μM, 50 μM, 75 μM, 100 μM) were prepared in the laboratory. Photos of sodium fluorescein solution with standard concentrations were taken using the same equipment and algorithms in the experiment. The standard calibration curve in Fig. 5c was used to calibrate the fluorescence intensity value. As shown in Fig. 5c, the relative values of concentration gradient produced by the concentration gradient generator with bubble removal devices are 0, 0.22, 0.45, 0.88, and 1, which are nearly equivalent to the theoretical values of 0, 0.25, 0.5, 0.75, and 1.

### BBR method used in non-Newtonian liquids

**Fig. 6:**
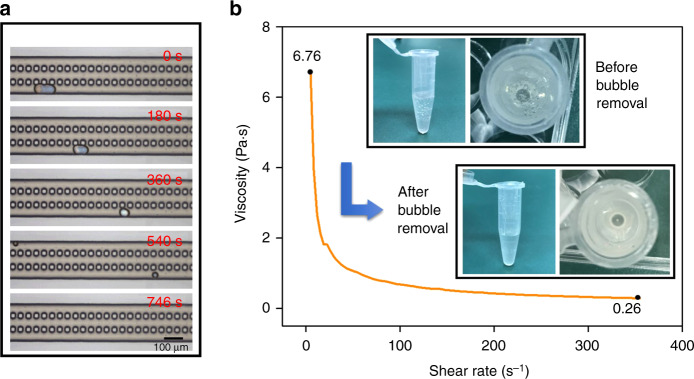
Bubble removal of carboxymethyl cellulose. **a** The bubble removal device could be capable of eliminating bubbles in a shear-thinning gel with a viscosity of 6.76 Pa s. **b** bubbles were removed from a shear-thinning gel

Non-Newtonian liquids with a high viscosity, such as gels, are widely used in the field of cell biology. Bubbles easily occur but are difficult to remove in such fluids. Any residual bubbles may have negative influences in experimental processes, especially when accurate control under a slow flow is required. To test the bubble removal capability of the proposed BBR method in fluids with high viscosity, a shear-thinning water-soluble binder (carboxymethyl cellulose) with viscosity of 6.76 Pa s was injected into the bubble removal device with a channel width of 85 µm and a flow rate of 152 nL/min. Similar to Newtonian fluids, gas bubbles can be effectively captured by pits and then eliminated gradually (Fig. 6a). Then, a bubble-free binder was obtained (Fig. 6b). This experimental result can well demonstrate the device’s capability of bubble removal in non-Newtonian fluids with high viscosity at a velocity of nanoliters per minute.

## Discussion

In summary, we have proposed a novel bioinspired bubble removal approach for microchannels based on the embolism repair mechanism of angiosperm. Without any auxiliary equipment, the new bioinspired method exhibits a prominent ability to remove bubbles while maintaining the stability and continuity of the flow. The proposed BBR method has the capability of removing bubbles in fluids with different viscosities and under a large range of flow rates (2 µL/min–850 µL/min), which indicates a wide range of applications. Simultaneously, the BBR method can not only eliminate bubbles in Newtonian liquids inside complicated structures (the case of a concentration gradient generator) but also successfully eliminate bubbles in non-Newtonian fluids with high viscosity (6.76 Pa s) at a velocity of nanoliter per minute (152 nL/min), although high-viscosity fluids have difficulty eliminating bubbles even in a vacuum. In addition, the BBR method was also characterized by high manufacturability, low cost, and easy implementation. The current BBR method reveals broad application prospects in macro/microfluidic fields ranging from 3D printing, implantable devices, virus detection, and biomedical fluid processing to microscale reactor operation and beyond.

## Materials and methods

### Plant material

Branches of Populus ussuriensis were collected from Dalian University of Technology, located northeast China. Plant material was brought to the laboratory in moist plastic bags and cut into slice samples of 10 cm length and 1 mm thickness. Samples were then fixed in FAA for 24 h and dehydrated in an alcohol series of 50, 70, 95, and 100% for 5 min, followed by lyophilization. Samples were fixed on aluminum stubs with double-sided electron conductive carbon cement and coated with gold. Samples were observed with a scanning electron microscope (SU8220, Hitachi High Technologies Corp., Tokyo, Japan) at an accelerating voltage of 5 kV.

### Numerical approach

To analyze the bubble removal process in the xylem conduit of angiosperm and verify the proposed mathematical model, a numerical simulation was conducted by using ANSYS FLUENT software. The CFD computational model is shown in Fig. S1a. The geometric model has three parallel conduits and rows of pits to interconnect them. It was constructed at the same scale as the vessel structure of the angiosperm xylem. The simulation conditions were set as pressure-implicit with splitting of operators (PISO). Mesh evaluation was performed to ensure that the results were not mesh-dependent.

### Fabrication of the bubble removal device based on the BBR method

A 1 mm-thick silicon wafer was chosen as the bottom substrate, and the thickness of the glass cover was 1 mm. The microstructures on the bottom substrate were fabricated by a deep reactive iron etch technique (Fig. S2a). As shown in Fig. S3, to study the bubble removal effect of the device, 10 mm-long microchannels were fabricated with a height of 30 µm and widths from 55 µm to 110 µm. To study the influence of the size and distance of pits on removal time, pits with sizes from 20 µm to 50 µm and distances from 20 µm to 100 µm were fabricated. A T-junction channel was integrated into the bottom substrate to generate bubbles. The inlet and outlet on the glass cover were fabricated by ultrasonic drilling. After cleaning, the glass cover and bottom substrate were sealed by an anode bonding process. Figure 2e shows a photograph of an assembly bioinspired bubble removal device.

### Measuring equipment

In this study, the temperature of the solution and the surroundings was 25 °C. A precision syringe pump (70–2001, Harvard, America) equipped with medical plastic syringes (10 mL, ZYMM, China) was used as the water injection system. A constant pressure pump (WH-PMPP-15, Wenhao Co., China) was used to control the gas flow. All of these devices were connected by polytetrafluoroethylene (PTFE) pipes. The testing setup for the evaluation of bubble removal was built with a measurement microscope (STM6-F10-3, Olympus, Japan), two pressure sensors (Fig. S4), a data-acquisition card, and a computer. The bubble area and bubble removal rate were obtained by image analysis using Image Pro-plus software. Data in this paper are presented as the mean ± standard deviation (SD).

### Measure of viscosity

To evaluate the effect of viscosity on bubble removal, carboxymethylcellulose sodium (CMC-Na) was added to deionized water as a thickening agent. The concentrations were 0.2 mg/mL, 0.4 mg/mL and 0.6 mg/mL, and the corresponding viscosities were 65.38 mPa s, 75.62 mPa s and 93.57 mPa s at 25 °C, respectively (measured by a rheometer (DSR 500, LAMY, France)).

### Fluorescence test

To demonstrate the effectiveness of the BBR method for complex microfluidic devices, a concentration gradient generator was fabricated based on a soft etching process. The SU-8 mold was fabricated by a photolithography technique (Fig. S2b). The SU-8 mold was finished by assembling the glass substrate and an aluminum frame, which was used to pour liquid polydimethylsiloxane (PDMS). Liquid-phase PDMS mixed by base and curing agent in a 10:1 ratio (Sylgard 184, Dow Corning, USA) was poured into the SU-8 mold and degassed in a vacuum oven (HMDS-2, CETC, China) without being heated for 5 min. Then, it was baked at 85 °C for 1.5 h. After being cured, PDMS was peeled off from the SU-8 mold to obtain the device. A fluorescence microscope with a digital camera (IX71, Olympus, Japan) was used for the concentration test.

## Supplementary information

A bioinspired bubble removal method in microchannels based on xylem embolism repair of angiosperm

Bubble removal ability of the BBR method

Bubble’s behavior in microchannel without pits

Bubble removal process when impurities or particles are trapped in the channel

Bubble removal process in the device with channel numbers of 3

## Data Availability

The data that support the findings of this study are available from the corresponding author upon reasonable request.
