# Supplementary material for: A bioinspired bubble removal method in microchannels based on angiosperm xylem embolism repair
Source: Microsyst Nanoeng. 2022 Mar 22;8:34. doi: 10.1038/s41378-022-00367-1 (PMC8940964; doi:10.1038/s41378-022-00367-1)
Supplement: Supplementary file 1 — A bioinspired bubble removal method in microchannels based on xylem embolism repair of angiosperm [file 41378_2022_367_MOESM1_ESM.docx]

**A bioinspired bubble removal method in microchannels** **based on xylem embolism repair of angiosperm**

**Lihua Guo^1^, Yuanchang Liu^2^, Penghui Ran^1^, Gang Wang ^1^, Jie Shan^1^, Xudong Li^1^, Chong Liu^1^, Jingmin Li^1^***

1 Key Laboratory for Micro/Nano Technology and System of Liaoning Province, Dalian University of Technology, Dalian, China

2 Department of Mechanical Engineering, University College London, Torrington Place, London WC1E 7JE, UK

[guolihuas@163.com](mailto:guolihuas@163.com), [yuanchang.liu@ucl.ac.uk](mailto:yuanchang.liu@ucl.ac.uk), [ranpenghui@mail.dlut.edu.cn](mailto:ranpenghui@mail.dlut.edu.cn), [g21904060@mail.dlut.edu.cn](mailto:g21904060@mail.dlut.edu.cn), [ShanJie@mail.dlut.edu.cn](mailto:ShanJie@mail.dlut.edu.cn), [lixudong2015@mail.dlut.edu.cn](mailto:lixudong2015@mail.dlut.edu.cn), [chongl@dlut.edu.cn](mailto:chongl@dlut.edu.cn), [jingminl@dlut.edu.cn](mailto:jingminl@dlut.edu.cn)

* Corresponding author, [jingminl@dlut.edu.cn](mailto:jingminl@dlut.edu.cn)

**Detailed comparison of different bubble removal methods**

At the moment, dominant approaches of bubbles removal in microchannels include the method of increasing inlet pressure, method of bubble displacement with flow, method of integrating bubble trap structures, gas-liquid separation method based on surface treatment and bubble extract method based on hydrophobic porous membrane.

**The method of increasing inlet pressure** is realized by temporarily sealing the device’s outlet and increasing inlet pressure or speeding up flow rate simultaneously^5^. But the method could only be carried out before the start of the experiment. For the bubbles that enter the device during the experiment, increasing pressure or flow rate could destroy the flow stability and lead to experiment failure, especially when microfluidic devices are used as disposable products, such as point-of-care devices, cell culture devices and water quality detection devices. In these applications, conventional degassing methods are not applicable. For example, in cell culture devices, it always takes several days or weeks to supply the culture medium continually for cells. In this long period, air bubbles will be introduced from the entrance inevitably. If inlet pressure or flow rate is high, it will threaten cell viability and culture efficiency in microfluidic cell culture devices.

**Method of bubble displacement with flow** can be obtained by flushing microchannels with low-polarity aqueous solutions, such as ethanol and surfactants^6,7^, and treating surface with hydrophilic modification methods^4,8^. Pressure pulses are also helpful^5^. But these methods can only be carried out before the start of the experiment. It’s cannot be used for the bubbles that enter the device during the experiment.

**Method of integrating bubble trap structures**, such as sieve-like elements to captured bubbles or two coaxial tubes for bubble trapping^9-11^, have the drawback of limited trapping volumes. For example, the maximum bubble trap volume given in the literature^9^ is 10 µL, which will be disable to trap bubbles when the bubble trap is full. Therefore, this method is not suitable for long time and large amount of bubble removal.

**Bubble extract method based on hydrophobic porous membrane.** This method has the advantages of high bubble removal rate, but there are some drawbacks. **Firstly**, **it has limited application.** The bubble extract method cannot be used in liquid with high viscosity, liquid with high pressure or high flow rate or gas impermeable material. High viscosity fluids have a thick boundary layer that prevents gas permeating through the membrane. The bubble extract method is not suitable for bubble removal of high viscosity fluids. In addition, pressure and flow rate that is higher than a critical value will cause liquid leakage from porous hydrophobic membrane ^19^. What’s more, the bubble extract method relies on the hydrophobicity of material to trap bubbles and the gas permeability of material to extract air bubbles from liquid. It cannot be used in the situation where hydrophilic modification of the microchannel wall is required and the situation where microfluidic chips are used in liquid environment, such as implantable devices, water quality detection devices. **Secondly, it is difficult integration.** Porous hydrophobic membrane usually connects to the device by double-sided tape or multilayered structure. Poor manufacturing accuracy of double-sided tape could affect the performance of the device. In addition, the multilayered structure is relatively difficult to manufacture and integrate. To improve the efficiency of bubble removal, vacuum need to be created at the other side of the permeable material, but vacuum equipment is not portable and not inconvenient to integrate.

Compared to the characteristics and performance of the existing bubble removal methods, the BBR method proposed in this work has the following advantages. **Firstly, it has wide range of applications.** The BBR method can remove bubbles in both high viscosity fluids and regular fluids. It could also remove bubbles in liquid with high pressure (when dealing with high viscosity fluid) and high flow rate (by increasing channel number and adjusting structural parameters). The BBR method can adjust bubble removal rate and bubble removal amount to meet the requirements of different environments by changing its geometric parameters and channel number. Thus, the BBR method has a wide range of applications in microfluidic field, including removing bubbles in high viscosity liquid (such as gels), removing bubbles in the situation where hydrophilic modification of the microchannel wall is required and the situation where microfluidic chips is used in liquid environment (such as implantable devices, on-line water quality detection devices). **Secondly**, **the BBR method is not permeable material dependent and has no material restrictions.** The BBR method doesn’t rely on the hydrophobicity of material to trap bubbles and the gas permeability of material to extract air bubbles. BBR method removes bubbles based on the bionic mechanism that resides in xylem conduits. Bubbles are captured by pits and dissolved in liquid gradually by using the pressure difference generated around the captured bubble. Therefore, there is no limitation for the material to fabricate the device by using this method. BBR method can be used for both hydrophobic materials (such as PDMS and PMMA), and hydrophilic materials (such as silicon and glass). **Thirdly, the BBR method is easy to be fabricated and integrated.** The bioinspired bubble removal unit has two layers, and only one layer is needed to be photolithographed. In addition, this method could remove bubbles without additional vacuum equipment. It is easy to fabricate and integrate into various devices according to the application requirements. **Fourthly, the BBR method could remove bubbles in an automatic, real-time and steady way.** The BBR method can remove bubbles automatically and continuously in real-time without the aid of human interference and external equipment.

A detailed comparison of different bubble removal methods is summarized in Table S1.

**Table S1 Comparison of the advantages of the method in our work with similar studies.**

| **Mechanism** | **Method** | $\boldsymbol{v}_{\boldsymbol{av}}$ | $\boldsymbol{Auto}$  $\boldsymbol{\&}$ **Rt** | **Liquid** | **Substrate** | **Layer** | **Disadvantage** | **Ref.** |
| --- | --- | --- | --- | --- | --- | --- | --- | --- |
| Increasing inlet pressure | Close outlet and increase pressure. | / | No | Water | PDMS | 2, plasma bonding | Not applicable during device’ operation and incapacity in dealing with bubbles entering with flow. | 5 |
| Bubble displacement with flow | Flush channel with ethanol | / | No | Water | PDMS | 2, plasma bonding |  | 5 |
| Bubble displacement with flow | Flush channel with culture medium | / | No | Culture medium | PDMS | 3, plasma bonding |  | 7 |
| Integrating bubble trap structures | Two coaxial tubes to trap bubbles | / | Yes | Water | PVC, Glass | 3, mechanical connection | Limited trapping volumes, difficult to integrate. | 10 |
| Integrating bubble trap structures | Sieve-like elements to trap bubbles | / | Yes | Water | PDMS | 2, Plasma bonding | Limited trapping volumes | 11 |
| Integrating bubble trap structures | Large chamber to trap bubbles while vacuum to degas | 2.3 µL s^−1^ | Yes | Water, ethanol, MOPS | PDMS, glass | 2, plasma bonding | Additional equipment required | 9 |
| Bubble traps with permeable materials | Micropump and hydrophobic porous membrane to degas | / | Yes | Whole blood | PMMA, PDMS, polycarbonate microporous membrane | 6, double-sided tape | Additional equipment required and difficult to integrate. | 15 |
| Bubble traps with permeable materials | Hydrophobic membrane to form closed valve | 60  µL/s/mm^2^ | Yes | Water, PBS | PTFE, PMMA | 3, double-sided adhesive tape | Adequate pressure needed | 16 |
| Bubble traps with permeable materials | Hydrophobic surfaces to transport bubbles while membranes to degas | / | Yes | H_2_SO_4_, NaHCO_3_ | Silicon, PTFE porous membrane, acrylic plate | 3, double-sided tape | Liquid leakage, external gas entry, limited application and difficult to integrate | 18 |
| **Table S1 (Continuation) Comparison of the advantages of the method in our work with similar studies.** | | | | | | | | |
| **Mechanism** | **Method** | $\boldsymbol{v}_{\boldsymbol{av}}$ | $\boldsymbol{Auto}$  $\boldsymbol{\&}$ **Rt** | **Liquid** | **Substrate** | **Layer** | **Disadvantage** | **Ref.** |
| Bubble traps with permeable materials | Hydrophobic membrane sticks to channel | 444µL/s | Yes | Water | PMMA, porous hydrophobic membranes | 3, double-sided tape | Liquid leakage, external gas entry, limited application | 19 |
| Bioinspired bubble removal method | Bioinspired structures to trap and remove bubbles | 560 µL/s | Yes | Newtonian and non- Newtonian liquids | Permeable and impermeable materials | 2, anodic bonding | / | Our work |

$v_{av}$: Average bubble removal rate

$Auto\&\mathrm{Rt}:$ Bubble removal in automatic and real-time way

Layer: The layer numbers and bonding method adopted to fabricate bubble removal device.

MOPS: Biological buffer solution


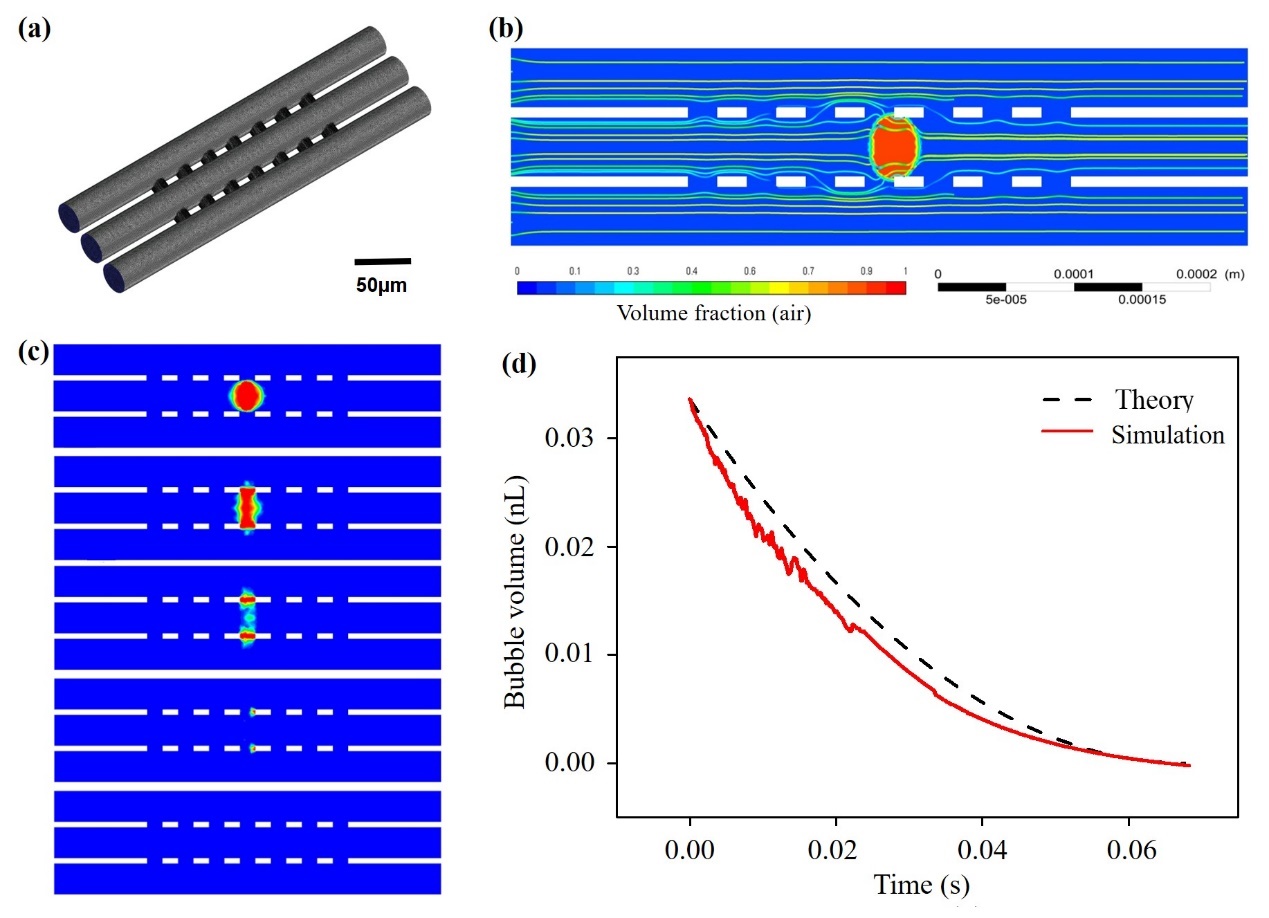


**Fig. S1 Numerical simulation of bubble removal in xylem vessel.** **a.** The CFD computational model. **b.** Water pathlines when an air bubble blocks the channel. **c.** Bubble removal process. **d.** Bubble removal time of simulation and prediction.


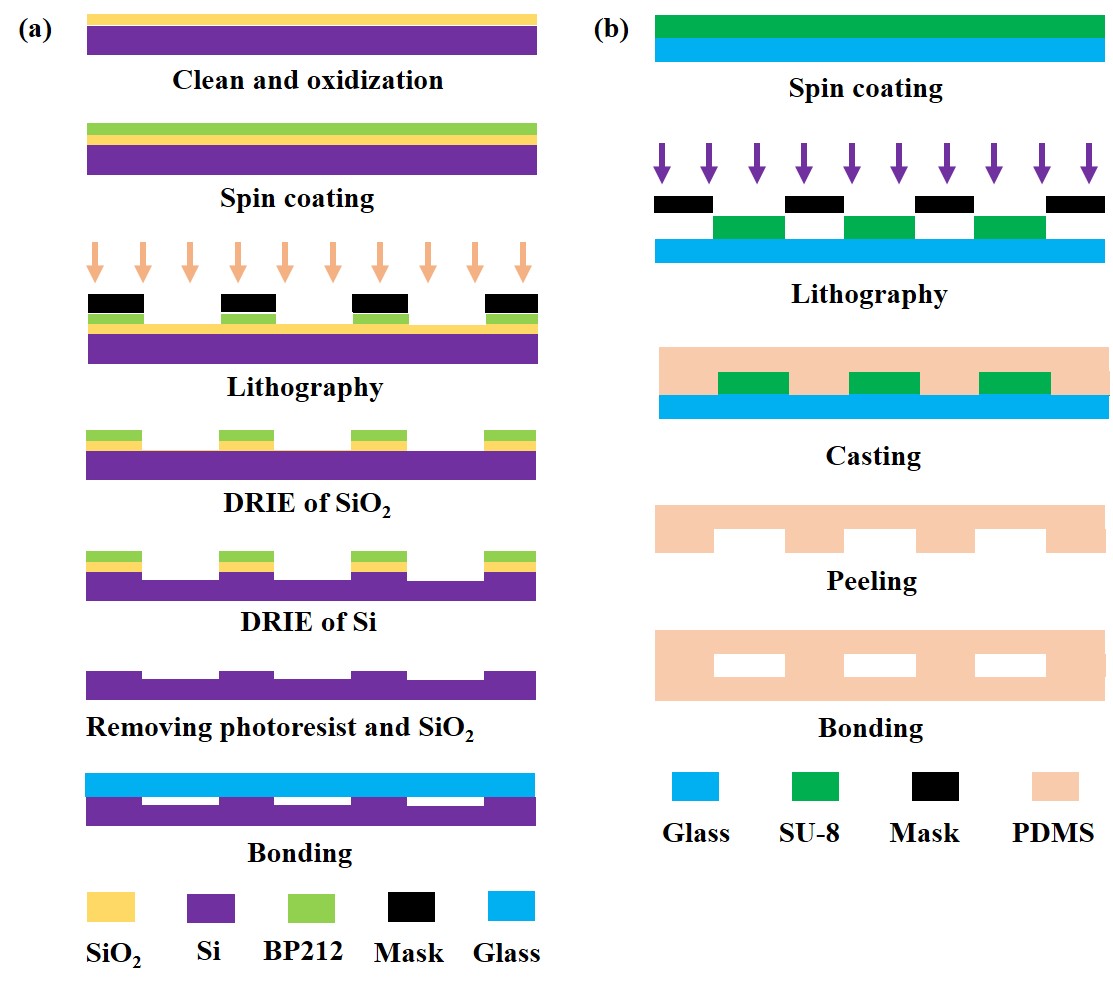


**Fig. S2 The fabrication process of the bubble removal device and the concentration gradient generator. a.** The dry etching process of fabricating bubble removal device. **b.** The soft etching process of fabricating concentration gradient generator.


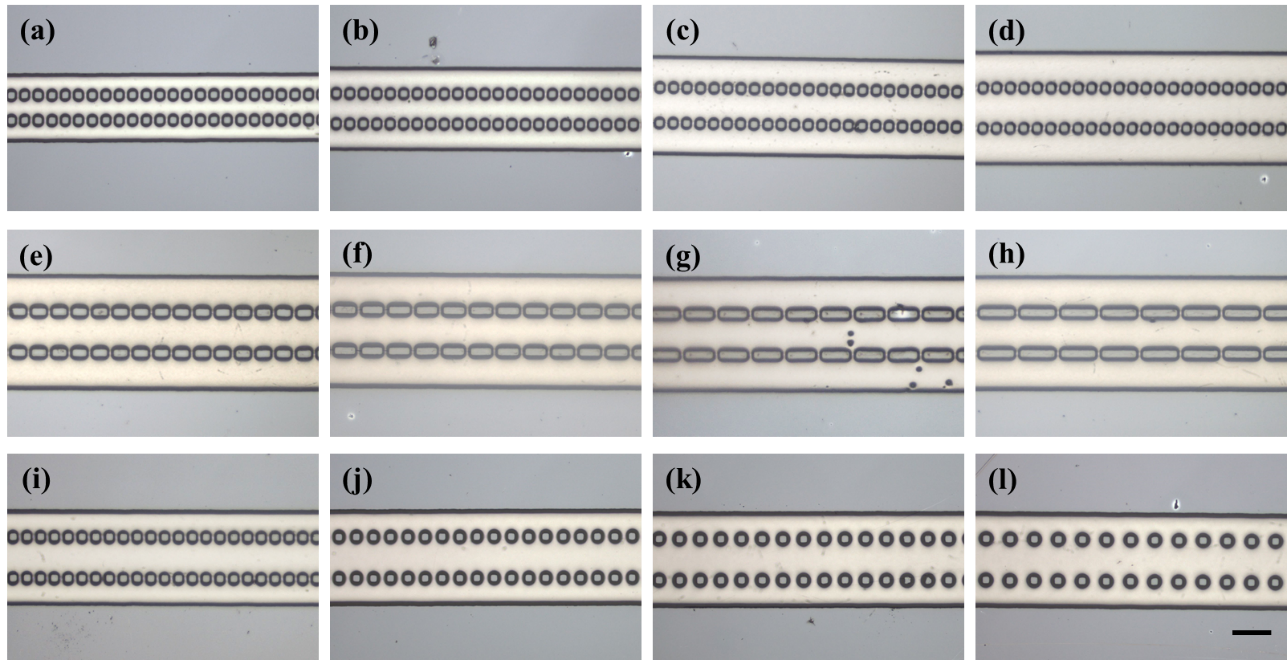


**Fig. S3 A series of bubble removal devices based on the BBR method.** **a-d.** Microchannels with width from 55 µm to 110 µm. **e-h.** Microchannels with pit distance from 40 µm to 100 µm. **i-l.** Microchannels with pits size from 20 µm to 50 µm.


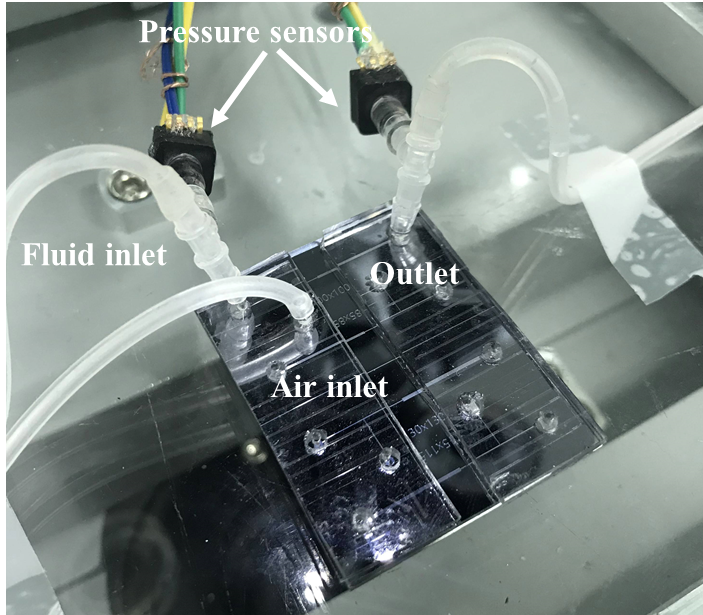


**Fig. S4 Two pressure sensors used for evaluating bubble removal performance.**


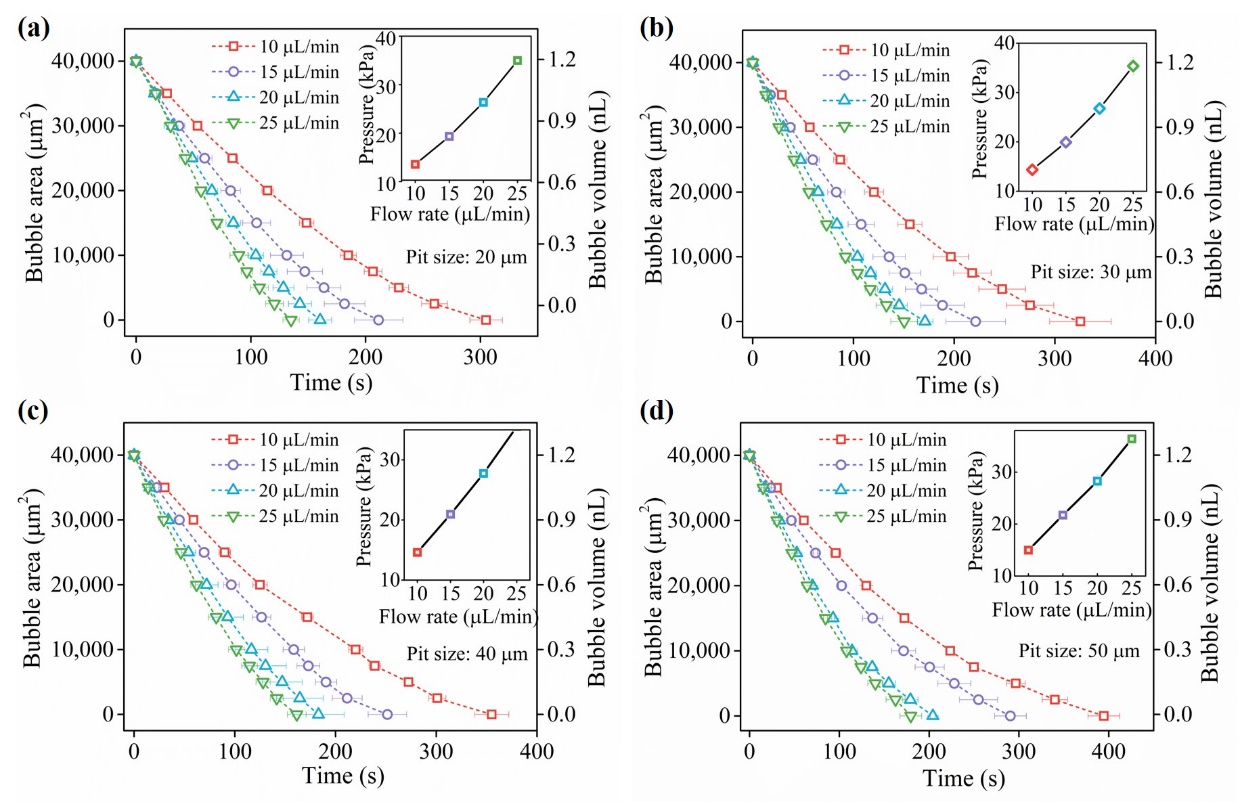


**Fig. S5 Removal performance of the bioinspired bubble remover with different pit size and flow rate.** **a.** Pit size is 20 µm. Variation of the bubble removal time (135±7 s, 160±10 s, 211±21 s, 305±14 s) with respect to flow rate of 10 µL/min, 15 µL/min, 20 µL/min and 25 µL/min. **b.** Pit size is 30 µm. Variation of the bubble removal time (150±13 s, 171±8 s, 221±29 s, 325±30 s) with respect to flow rate of 10 µL/min, 15 µL/min, 20 µL/min and 25 µL/min. **c.** Pit size is 40 µm. Variation of the bubble removal time (161±9 s, 183±25 s, 251±19 s, 355±17 s) with respect to flow rate of 10 µL/min, 15 µL/min, 20 µL/min and 25 µL/min. **d.** Pit size is 50 µm. Variation of the bubble removal time (179±12 s, 204±3 s, 290±17 s, 394±17 s) with respect to flow rate of 10 µL/min, 15 µL/min, 20 µL/min and 25 µL/min.


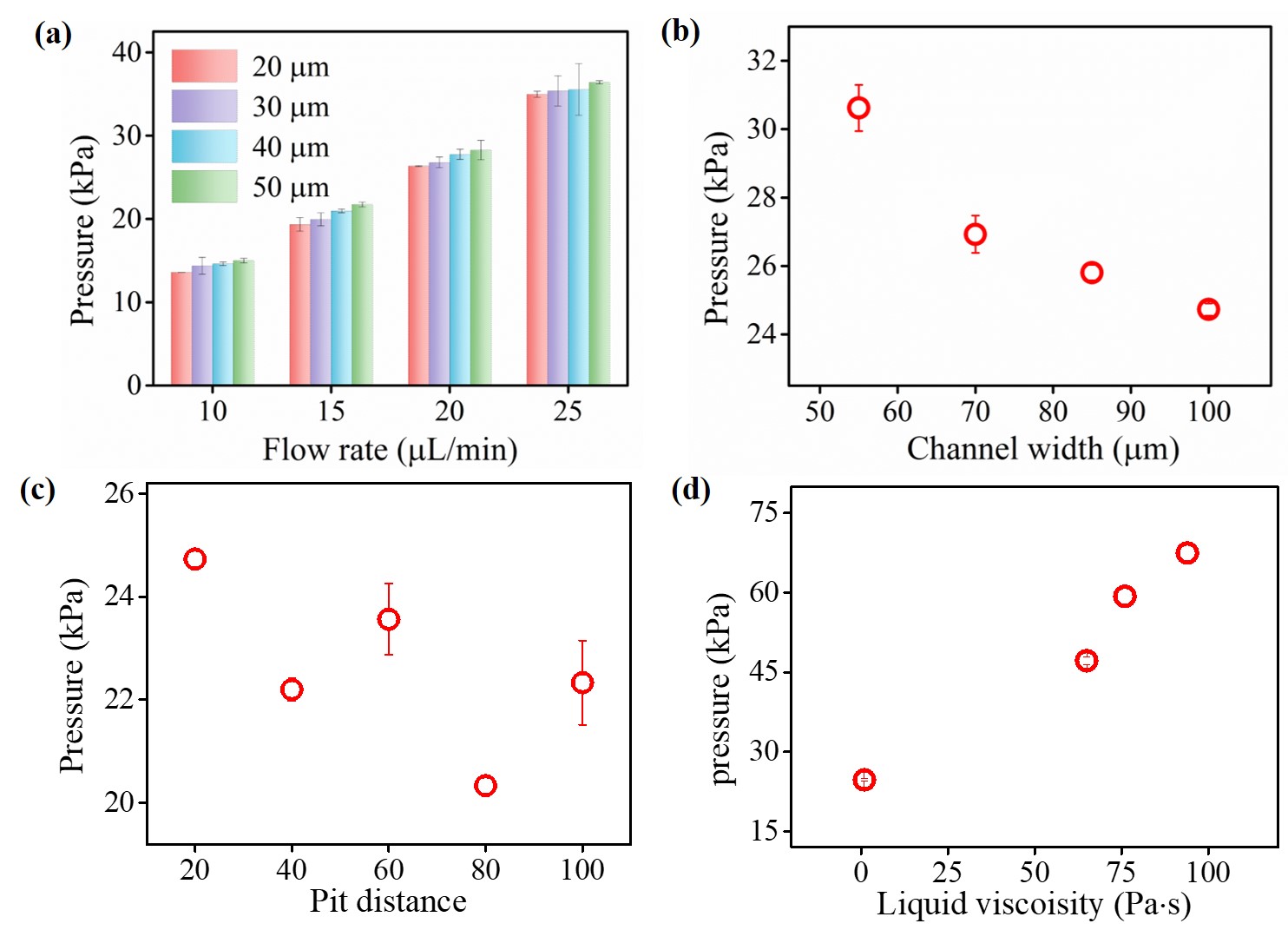


**Fig. S6 Pressure in the bubble removal process. a.** Variation of pressure with respect to the flow rate (10 µL/min, 15 µL/min, 20 µL/min, 25 µL/min) and pit size (20 µm, 30 µm, 40 µm, 50 µm). **b.** Variation of the pressure with respect to channel width of 55 µm, 70 µm, 85 µm and 100 µm (pit size of the bubble removal device is 20 µm, pit distance is 20 µm and flow rate is 15 µL/min). **c.** Variation of the pressure with respect to the pit distance (20 µm, 30 µm, 40 µm, 50 µm) with flow rate of 15 µL/min. **d.** Variation of the pressure with respect to the liquid viscosity (1 mPa·s, 65.38 mPa·s, 75.62 mPa·s and 93.57 mPa·s at 5 s^-1^,25 ℃, respectively).


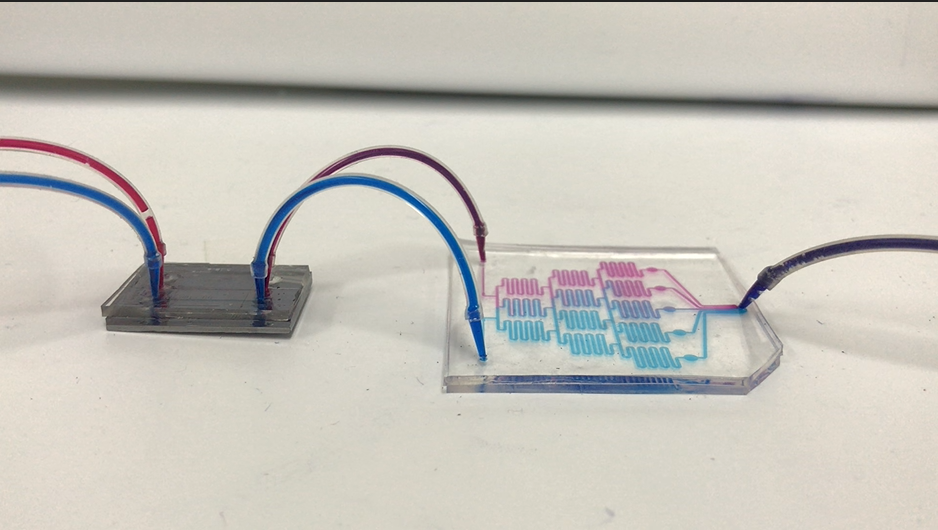


**Figure S7 A concentration gradient generator connected with a bubble removal device.**


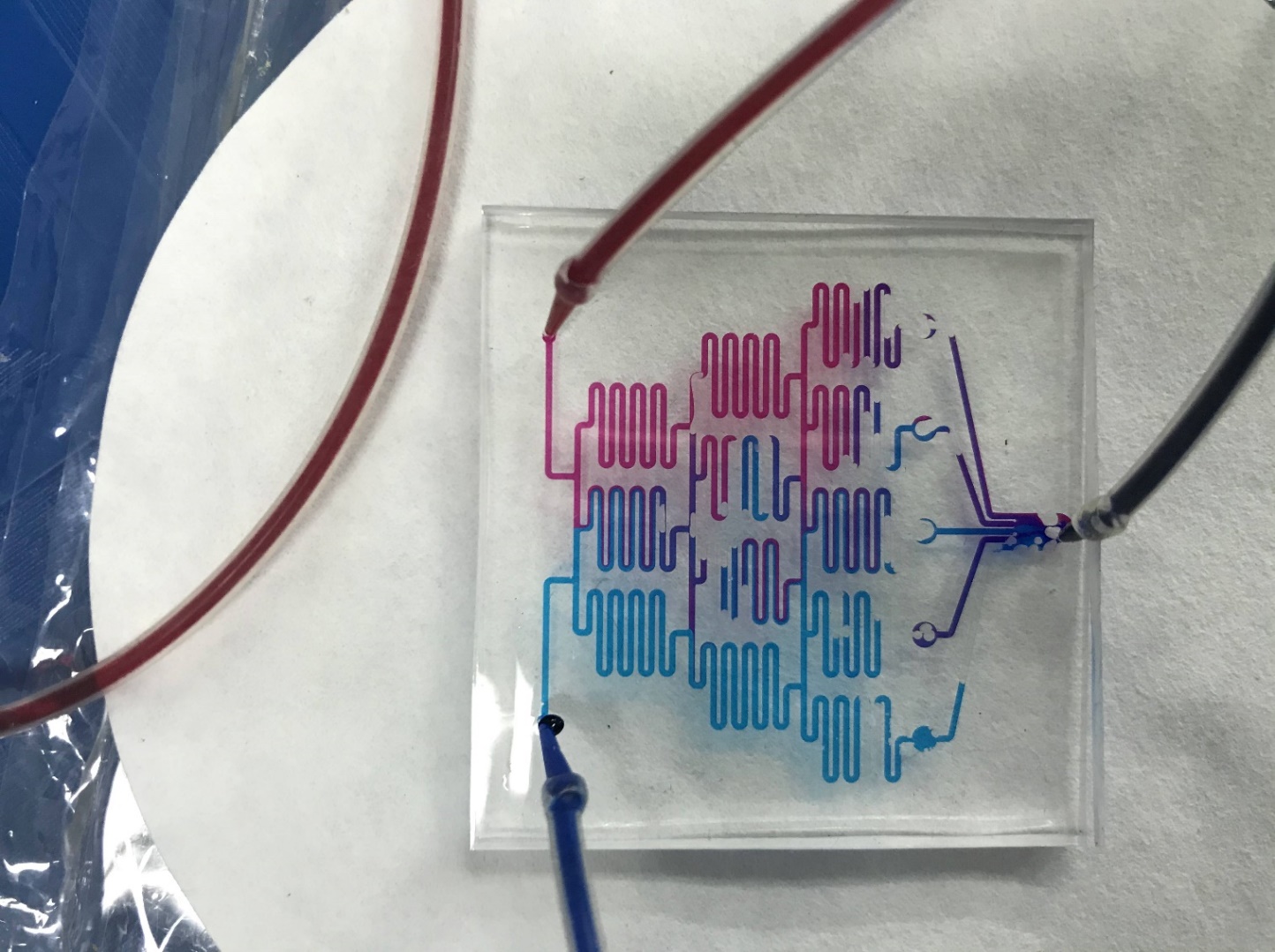


**Fig. S8 A concentration gradient generator without bubble removers.** Injected bubbles were flushed out or stuck and microchannels were blocked, which results in failure of concentration gradient generation.
